# Automated tumor analysis for molecular profiling in lung cancer

**DOI:** 10.18632/oncotarget.4391

**Published:** 2015-08-03

**Authors:** Peter W. Hamilton, Yinhai Wang, Clinton Boyd, Jacqueline A. James, Maurice B. Loughrey, Joseph P. Hougton, David P. Boyle, Paul Kelly, Perry Maxwell, David McCleary, James Diamond, Darragh G. McArt, Jonathon Tunstall, Peter Bankhead, Manuel Salto-Tellez

**Affiliations:** ^1^ Centre for Cancer Research and Cell Biology, Queen's University Belfast, Belfast, UK; ^2^ Department of Cellular and Molecular Pathology, Antrim Area Hospital, Antrim, UK; ^3^ Institute of Pathology, Royal Victoria Hospital, Belfast, UK; ^4^ PathXL Ltd, Northern Ireland Science Park, Belfast, UK

**Keywords:** molecular pathology, manual macrodissection, percentage tumor, image analysis, digital pathology

## Abstract

The discovery and clinical application of molecular biomarkers in solid tumors, increasingly relies on nucleic acid extraction from FFPE tissue sections and subsequent molecular profiling. This in turn requires the pathological review of haematoxylin & eosin (H&E) stained slides, to ensure sample quality, tumor DNA sufficiency by visually estimating the percentage tumor nuclei and tumor annotation for manual macrodissection. In this study on NSCLC, we demonstrate considerable variation in tumor nuclei percentage between pathologists, potentially undermining the precision of NSCLC molecular evaluation and emphasising the need for quantitative tumor evaluation. We subsequently describe the development and validation of a system called *TissueMark* for automated tumor annotation and percentage tumor nuclei measurement in NSCLC using computerized image analysis. Evaluation of 245 NSCLC slides showed precise automated tumor annotation of cases using *Tissuemark*, strong concordance with manually drawn boundaries and identical EGFR mutational status, following manual macrodissection from the image analysis generated tumor boundaries. Automated analysis of cell counts for % tumor measurements by *Tissuemark* showed reduced variability and significant correlation (*p* < 0.001) with benchmark tumor cell counts. This study demonstrates a robust image analysis technology that can facilitate the automated quantitative analysis of tissue samples for molecular profiling in discovery and diagnostics.

## INTRODUCTION

Personalised medicine aims to stratify patients’ cancers into new molecular subtypes who can benefit from individualised therapy [1–3]. The translation and validation of new molecular biomarkers in cancer relies heavily on molecular pathology and the investigation of specific mutations or other genomic anomalies in nucleic acids extracted from formalin fixed, paraffin embedded (FFPE) human tissues.

**Figure 1:**
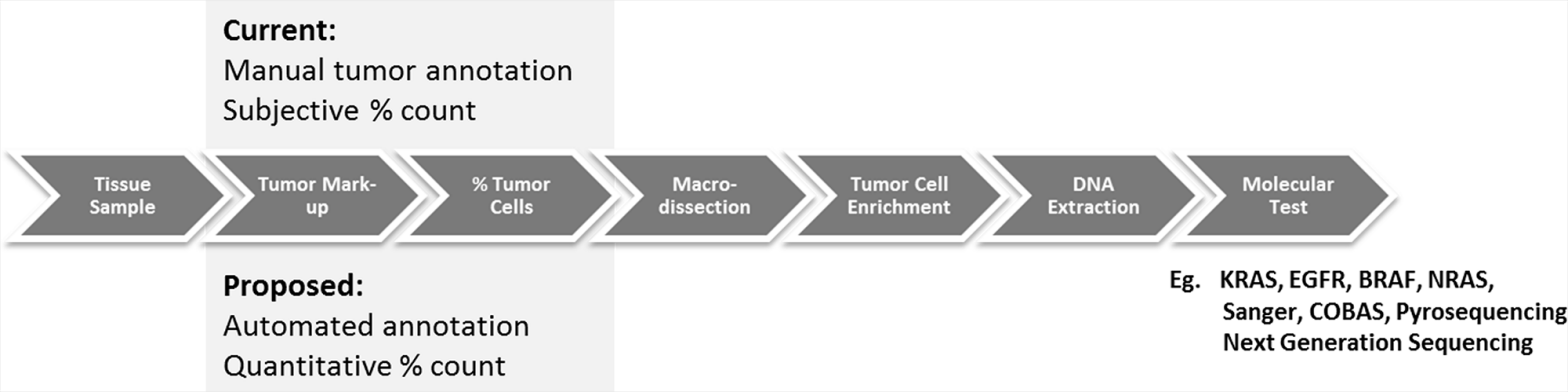
Comparison of current methods for macrodissection based on manual annotation (top) and the proposed automated tumor annotation for macrodissection (bottom)

Extracting DNA and RNA from tumor cells in the context of FFPE samples is not straightforward. Most tissues containing tumor also contain a mixture of cell types such as non-neoplastic epithelial cells, mesenchymal tissue, inflammatory cells and acellular material such as mucin which has an influence on subsequent processes including nucleic acid isolation, PCR amplification and next generation sequencing [4]. Therefore in most studies, it is important to determine the tumor nuclei content by visually estimating the percentage tumor cells and where that falls below a certain threshold, to enrich the tumor cell contents of the sample by manual macrodissection (Figure 1), to a) make the sample suitable depending on the sensitivity of the test, and b) make the molecular analysis as broad as possible to be able to identify “clonal disease”.

Macrodissection is a manual process in which the region of tumor is physically scraped from the slide using a scalpel into a receptacle for subsequent nucleic acid extraction and molecular analysis. Marking the regions of viable tumor within tissue samples relies on the visual assessment of haematoxylin and eosin (H&E) tissue sections, usually by an experienced pathologist and typically performed at low power magnification. Manual macrodissection aims to enrich the proportion of neoplastic cell nucleic acid by removing non-tumor containing regions, not only increasing the likelihood of detecting a relevant mutation if it exists but also increasing the certainty that a mutant signature originates from the malignant cell isolate. Percentage tumor evaluation and macrodissection underpin most of the new and emerging molecular tests for solid tumors including *RAS, EGFR, BRAF* mutational analysis, commercial tests such as Oncotype DX, Mammaprint, Prolaris and more recent clinical sequencing panels (e.g. Foundation One). Given that this role falls primarily to the pathologist, the rapid rise in the investigation of solid tumor molecular tests and their delivery in diagnostic molecular laboratories is putting a strain on pathology services. Molecular labs without in-house pathology competency, must transport slides to a qualified pathologist for review and manual annotation. The centralization of some molecular tests, despite having clear sample guidelines on their test request forms, must review the slides centrally and ensure sufficient tumor is present before macrodissection and analysis.

Whilst pathologists can usually confidently identify malignant cells within a tissue section, the tracing of boundaries for manual macrodissection is imprecise and inherently subjective. This has the potential of introducing inter-/intra-observer/laboratory variability and impacting the quality of molecular assays from the subsequently macro-dissected regions. This is particularly important in the evaluation of % tumor cells which can be highly subjective. For example, Smits *et al* [5] have shown significant variation in the reporting of tumor cell percentage across the same set of lung cancer samples. In a series of 47 cases, tumor cell percentage was overestimated in 45% of cases with only 14% of the observations being considered correct against precise cell counts on the same samples. Differences between pathologists could be as high as 40 points on the % scale for the same sample. Similar findings have been reported in colorectal cancer [6]. The demand for automation to support markup, high throughput analysis and precision is growing rapidly.

This is particularly important in lung cancer. Lung cancer is the number one cause of cancer-related deaths in both men and women, accounting for 1.38 million deaths a year in 2008 [7] with non-small cell lung cancer (NSCLC) accounting for approximately 87% of all lung cancer cases [8]. With an increasing understanding of how genetic mutations relate to cancer development, recent work has focused on the identification of molecular biomarkers for targeted molecular therapies [9]. A number of candidate molecular targets have been identified, including the human epidermal growth factor receptor (EGFR/HER/ERBB) family of receptors in humans (*EGFR* [10, 11], *HER2/neu* [12, 13], *HER3* [14, 15]), *RAS* [16, 17], *VEGF* [18], *ALK* [19], *MET* [8], *IGF1R* [20] and the *PI3K/Akt/mTOR* pathway [21]. Importantly, *EGFR* mutational status is vital in selecting patients for erlotinib and gefitinib therapy [22, 23]. As discussed, depending on the assay type, reliable *EGFR* mutational analysis requires significant numbers of tumor cells and frequently macrodissection to generate sufficient tumor DNA and avoid false negative test results.

With rapid developments in digital pathology, glass slides can now be digitised in their entirety at diagnostic resolution using whole slide scanning devices. This allows routine scanning of whole slides for the purposes of archiving, education, remote consultation and research [24–27]. It also provides the opportunity to develop and use computer based image analysis methods to automatically identify areas of tumor for macrodissected and quantitatively measure tumor cell percentage in H&E tissue samples. While there has been considerable effort in the image analysis community to develop quantitative immunohistochemistry (IHC), little work has been done using image analysis for H&E stained samples for the purposes of tumor identification and annotation. This study is the first to present a novel computerised approach for the automated identification of tumor regions and measurement of tumor percentage by cell counts from H&E stained tissue sections, with the specific goal of supporting recent advances in molecular pathology (Figure 1).

## RESULTS

### Inter-observer variability of tumor boundary annotation

**Figure 2:**
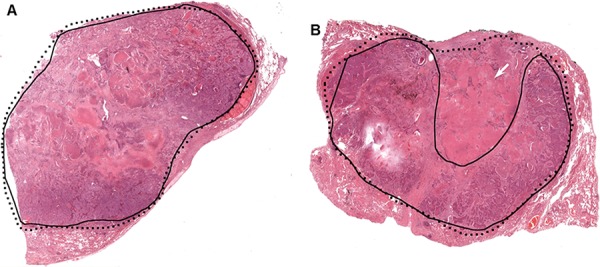
Two examples of manual tumor annotation showing observer varibility In both **A.** and **B.** the solid black contours were from the first review, with the dotted lines from the second review. A minor discrepancy exists between the two boundaries in (A) In (B), the larger discrepant area (shown with the white arrow) is a region of mixed tumor cells and necrotic tissue. This deviation might impact downstream molecular analysis.

During manual slide annotation, 15 randomly selected neoplastic tissue slides were annotated independently by two pathologists. Direct comparisons of the drawn boundaries on both occasions show overlap of tumour boundary between 68% and 98%. While most of the variations in manual annotation were considered minor, some samples showed important discrepancies which could have impacted in the estimation of tumor percentage and downstream molecular test results. Examples are shown in Figure 2.

### Inter-observer variation in percentage tumour cell estimates

**Figure 3:**
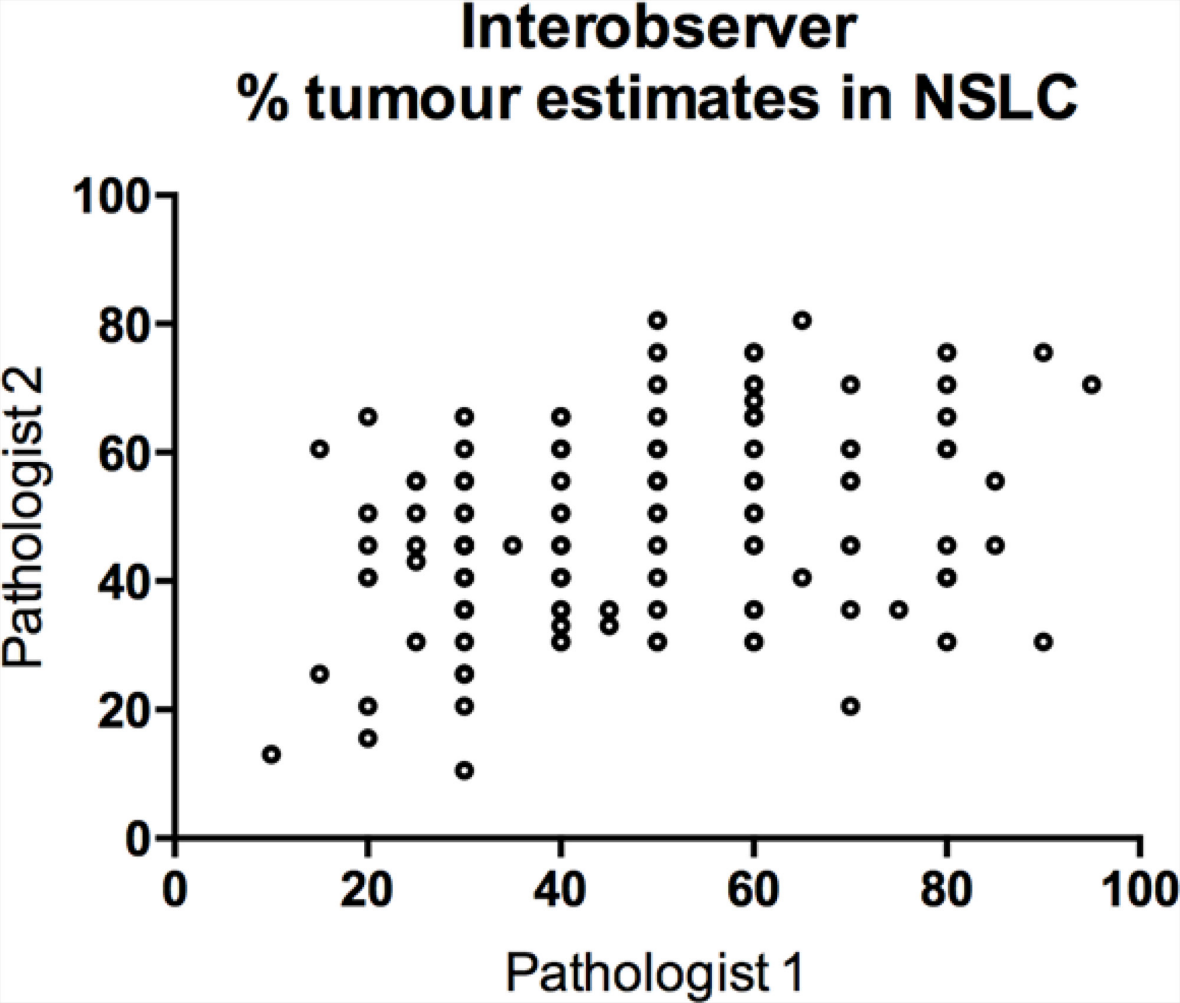
Scatterplot of tumor percentage for 136 lung cancer cases derived from two experienced pathologists, showing gross variation between estimates

The percentage of tumor was visually estimated independently by two pathologists for each of the 136 H&E stained slides containing tumor in this study, in the same way as is currently used in routine molecular diagnostics. There was a high degree of descepancy between the two pathologists’ estimations of % tumor (Figure 3). The Pearson's correlation coefficient *r* = 0.32 between the two reviews, indicated a poor correlation. In 51/136 cases (37%), the tumor percentage estimation discrepancy was greater than 20%. The largest difference showed a discrepancy of 60 units on the % scale (90% versus 30%). These results highlight the real inter-observer variation that exists in % tumor cell estimation in lung cancer samples.

**Figure 4:**
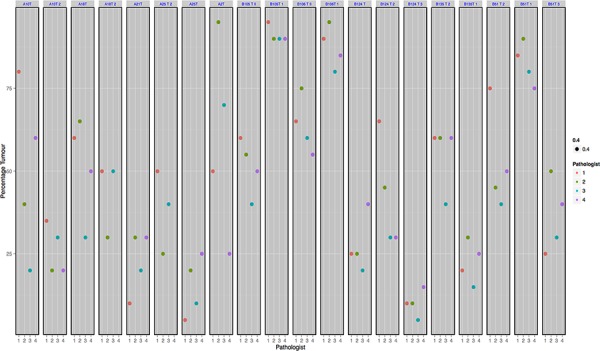
Shows the % tumor estimates provided by pathologists for 20 random regions of lung cancer Each column represents an indvidual case with color coded dots showing the % tumor estimates for each case.

In addition, a series of 20 H&E stained lung images were also circulated to four different pathologists for review and evaluation of percentage tumor. Tumor cell estimates by pathologists across the range of sample images are shown in Figure 4. The first column represents a single case reviewed by four pathologists with % estimates ranging from 20% to 80%. The mean maximum deviation across all cases was 25%. The pathologists are colour coded and on review did not show any consistent bias with regards to higher or lower scoring of percentage tumor cells. Again this illustrates the considerable variation that exists in the reporting of tumor percentage for molecular analysis and the need to improve objectivity of this evaluation.

**Figure 5:**
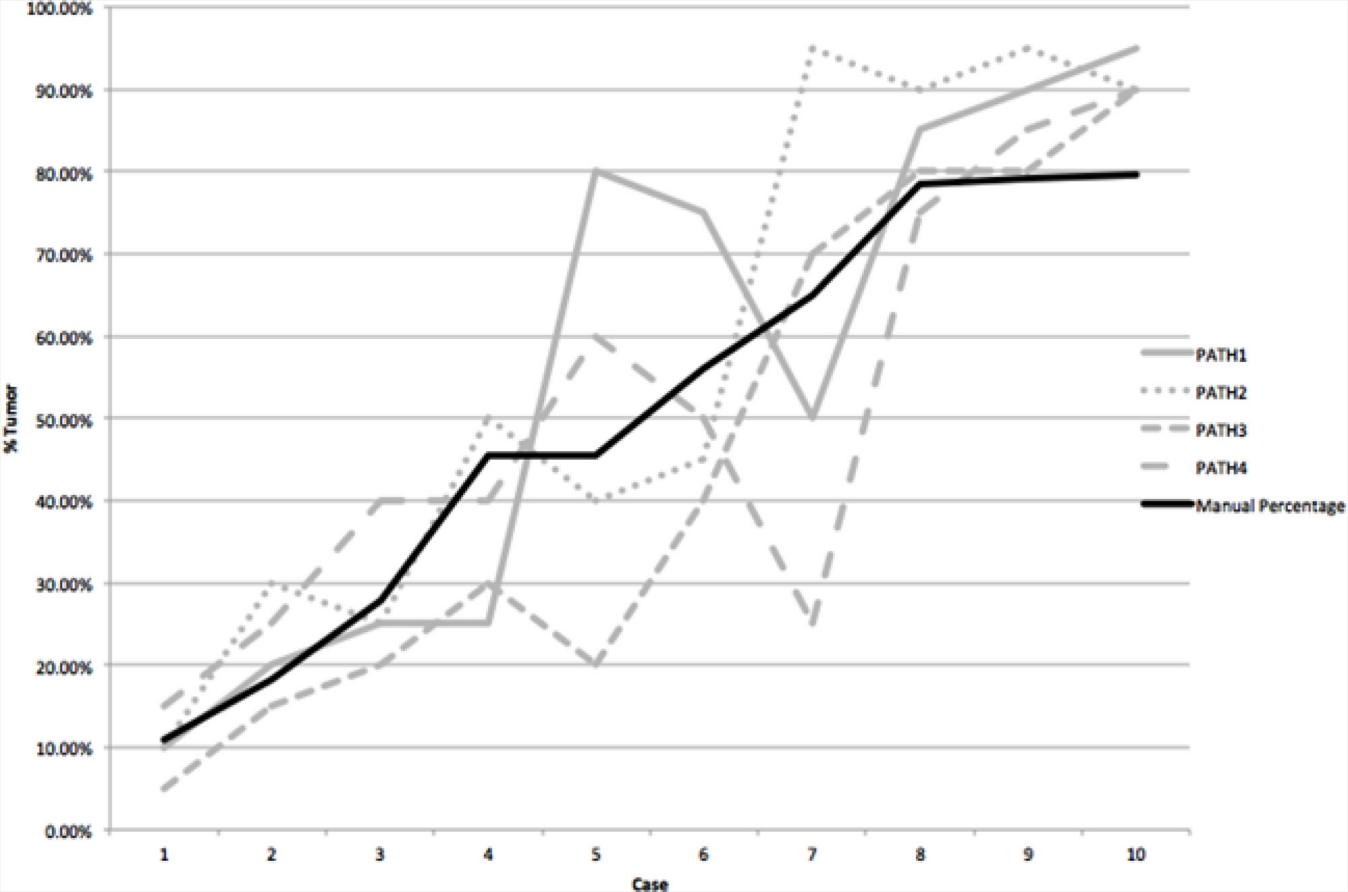
For ten cases the absolute numbers of tumor cells were counted This is shown by the solid black line for cases ordered by increasing % tumor cells. Visual estimation of % tumor cells by four pathologists is individually plotted as the grey lines. This shows no consistency in the overcalling or undercalling of % tumor cells.

Precise benchmark cell counts were available on 10 cases within this set. If a difference exceeding 10% from the benchmark number is considered unacceptable, only 50% of cases were considered correct. Comparison with benchmark data showed that pathologists overestimated % tumor in 32% of cases (Figure 5).

### Automated tissuemark calculation of tumour boundary

**Figure 6:**
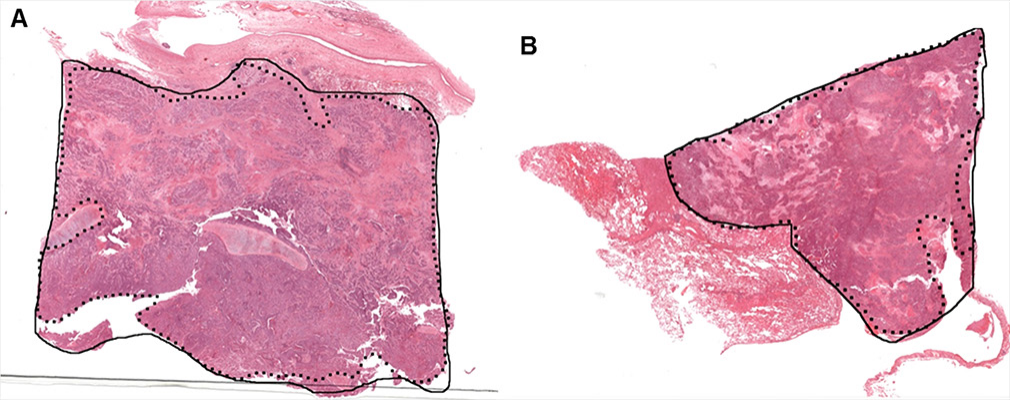
Two examples A. and B. showing the comparison between pathologist annotation (solid black line) with the automated macrodissection method (dotted black line) on H&E stained lung images Both examples show strong concordance between manual and automated boundaries.

Automated annotations were generated by *TissueMark* on the digital H&E images. Annotations generated by the pathologist and the automated *TissueMark* method were superimposed on all 136 slides containing tumor tissue. There was a high concordance between manual and automated tumor boundaries (Figure 6).

**Figure 7:**
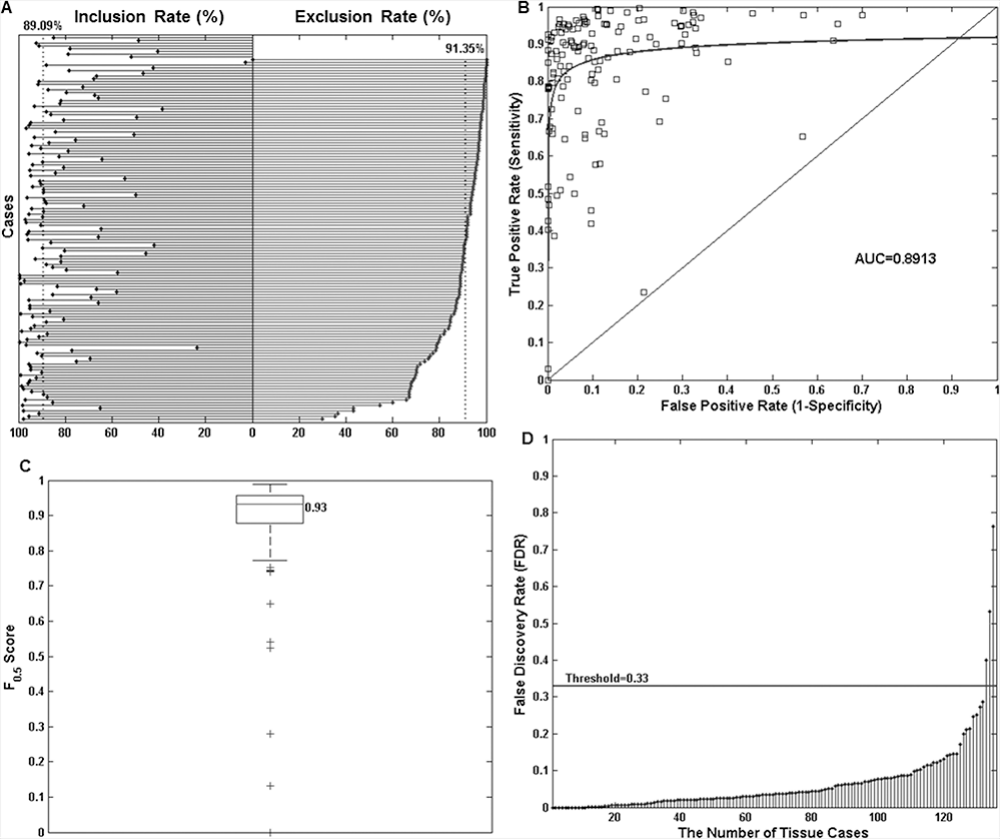
Objective comparison of manual and automated tumor annotations for 136 neoplastic lung tissue slides using four statistical measurements **A.** Shows the *inclusion rate* vs. *exclusion rate* plot. For each of the 136 slides, the *inclusion rate* figure (left) and *exclusion rate* figures (right) are shown. For clarity, this plot was sorted by *exclusion rate* in descending order. The two dotted vertical lines indicate the median *exclusion rate* value across all slides to be 91.70% and *inclusion rate* of 89.00%. The top 8 cases in the plot do not have *exclusion rate* values as the pathologist indicated that the entirety of these tissues should be taken forward for nucleic acid extraction without macrodissection. **B.** A receiver operating characteristic (*ROC*) curve shows the majority of cases achieved strong boundary concordance with low false positivity and high true positivity (sensitivity). The area under the curve (*AUC*) value is 0.89. **C.** A box plot for *CI* measurement. The median *CI* value is 0.93, and there are 10 outlier cases marked as “+” in the plot. **D.** A Stem plot of the false discovery rate (*FDR*) for all the cases. Using a threshold value of 0.33, results in only 3 outlier cases.

For statistical evaluation, it was necessary to measure boundary concordance using a variety of metrics as no single measurement was sufficient to reflect the range of differences that might be observed. The *inclusion rate* vs. *exclusion rate* plot is shown in Figure 7A. Results suggested the proposed automated tumor annotation approach is highly accurate with a median *exclusive error* rate of 91.70% and average *exclusive error* of 86.61%, and median *inclusive error* value of 89.00% and average *inclusive error* of 82.40%, accepting the pathologist's annotation as the gold standard. The *ROC* curve is shown in Figure 7B. It suggests that the majority of slides showed strong boundary concordance as most of the data points are located at the top left corner of the plot. The *AUC* also achieved the high value of 0.89. Figure 7C shows the CI presented as a box plot, with a high median CI value of 0.93 and 75% (102 out of 136) slides with a CI value higher than 0.88. The majority 92.65% (126 out of 136) slides achieved *CI* > 0.76 (the lower adjacent) which gave only 10 outlier cases as shown. On review, outlier cases were the result of weak staining or sparsely distributed tumor cells within the sample. *FDR* evaluation can be seen in Figure 7D where an average *FDR* of 0.06 was obtained. This is in the upper borderline range meaning that there is more chance that extracted nucleic acids will be from a neoplastic cell rather than a non-neoplastic cell. To give a safe margin of error, if we define the *FDR* acceptance standard to place twice as much importance on the identification of malignant cells than non-malignant cells, we get a threshold of *FDR′* = 0.33. At this threshold, 97.06% (132/136) of the slides were considered suitable for manual macrodissection.

For the 136 tumor slides, automated annotations were reviewed by an independent pathologist. The pathologist found good concordance between computer generated annotations and the manual annotations for a majority of cases with all automated slide annotations considered suitable for manual macrodissection. There were a small number of cases where the automated computer approach included small regions of non-neoplastic tissue within the annotation. Due to the small area of these regions, it was concluded by the molecular pathologist that in his subjective view, this would not affect the extraction of nucleic acids or subsequent sequencing analyses and that the automated annotations would ensure a sufficiently high percentage of malignant cells in the sample to carry out a successful molecular test.

For the 109 non-tumor slides, the automated method successfully identified 106 as non-tumor. Only 3 slides gave small regions of wrongly recognised tumor, which gave an overall success rate of 97.25%. These minor misclassifications were due to the presence of histologically benign areas including necrosis, pneumocyte hyperplasia and dense lymphocyte crowding due to inflammation.

### Heatmap generation

**Figure 8:**
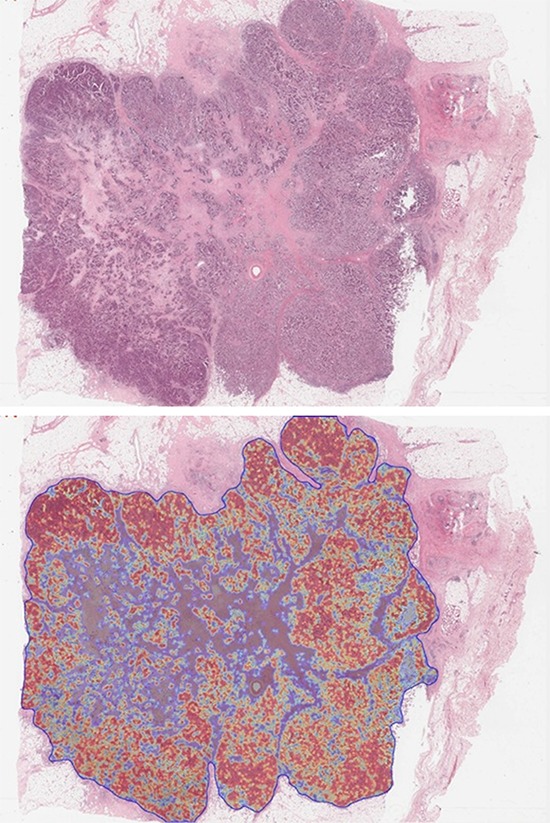
Illustrates how a *Tumormap* can be generated from the posterior probabilities, highlighting regions of high tumor probability (red) against low tumor probability (blue) and associated color spectrum within the generated tumor boundary

As each tile on the image is allocated a posterior probability in the range of 0–1 indicating its likelihood to be tumor or non-tumor, this can be visualised in the form a tumor heatmap (Figure 8). This *TumorMap* is a useful adjunct in assessing algorithm performance and the correct identification of tumour and non-tumour regions within the automatically annotated boundary.

#### Automated cell counting and tumour percentage

**Figure 9:**
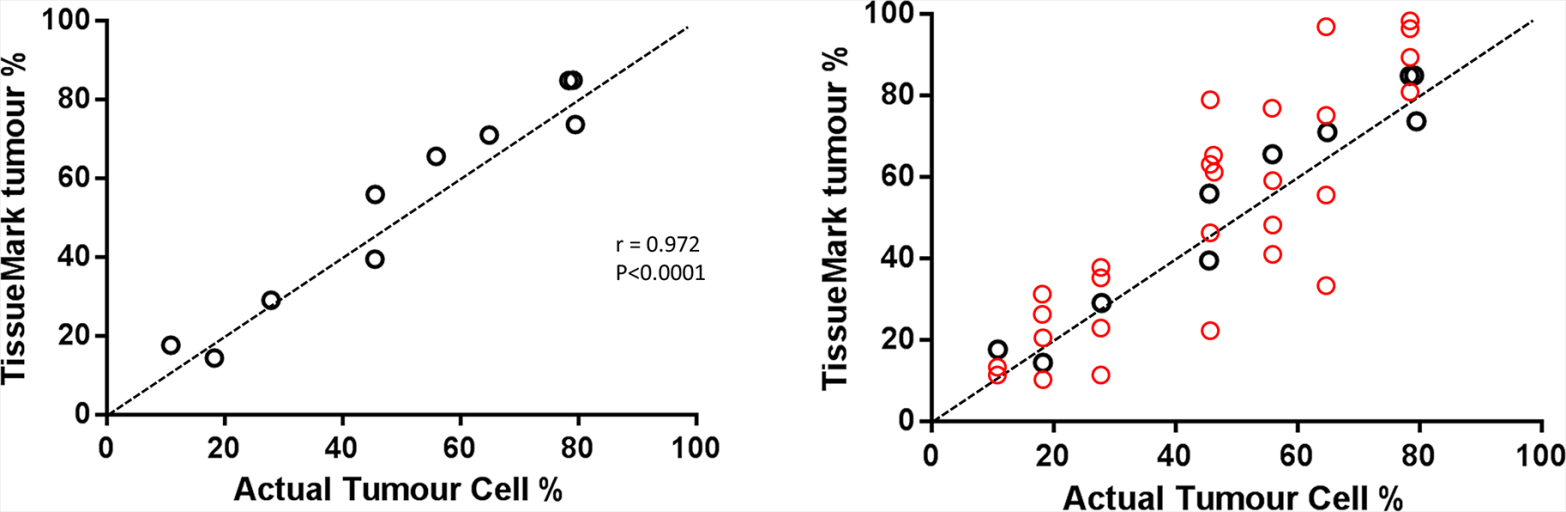
A. Comparison of automated tumour nuclei counts and percentage tumour values (y-axis), against benchmark data on tumor % showing strong correlation, mapping closely to actual tumor cell percentage values **B.** The same scatterplot as (A) but superimposing the range of pathology estimates (red circles) against the benchmark data.

Automated calculation of tumour percentage using TissueMark was compared against manual hand-counted results on a series of 10 images. A strong correlation (*r* = 0.972, *p* < 0.0001) was seen with all TissueMark results falling within 10 units of the benchmark (Figure 9A). This demonstrates that TissueMark analysis can rapidly estimate tumour percentage in tissue samples that closely correlates with the actual percentage. This provides much more consistent and repeatable results than seen for pathologist scores as shown in Figure 9B which show considerable variation around the benchmark values.

### Molecular evaluation

For the six cases evaluated, automated annotation generated precisely the same EGFR mutation results as conventional manual hand drawn annotations (Table 1). In addition, the DNA extracted following the automated identification had a similar 260/280 reading and, interestingly, an improved concentration yield in 5 out of 6 cases.

**Table 1:** Shows the six cases, macrodissected and assayed twice for EGFR, once using conventional manual annotation and once using automated tumor recognition

Case No.	Manual			Automated		
	NA Conc'n	260/280	Mutation Result	NA Conc'n	260/280	Mutation Result
1	213.7	1.8	MUTATION EXON 21 L858R	253.2	1.88	MUTATION EXON 21 L858R
2	252.6	1.85	MUTATION NOT DETECTED	207.8	1.87	MUTATION NOT DETECTED
3	76.6	1.84	MUTATION NOT DETECTED	129.2	1.83	MUTATION NOT DETECTED
4	133.5	1.88	MUTATION EXON 21 L858R	187.2	1.87	MUTATION EXON 21 L858R
5	101.2	1.83	MUTATION NOT DETECTED	179.6	1.88	MUTATION NOT DETECTED
6	47.6	1.85	MUTATION EXON 19 DELETION	72.3	1.86	MUTATION EXON 19 DELETION

Results show typical difference in nuclear acid concentration but full consistency in mutation status between the two methods.

## DISCUSSION

There have been considerable advances made in the molecular pathology of solid tumours with an increasing armoury of nucleic acid based tests for single or multiple biomarker assessment, some of which are now essential for patient stratification and therapeutic selection. These include recognised tests such as *RAS, BRAF* and *EGFR* mutation detection, OncotypeDX, Mammaprint and a range of emerging next generation sequencing oncology panels which will further enhance the molecular profiling of tumors and the targeted treatment of cancer patients. However, due to the complexity and heterogeneity of tissue, the reliability of molecular profiling is strongly dependent on the pathological review of tumor samples together with the selection and enrichment of tumor DNA by macrodissection. Nevertheless, pathological review, markup and assessing tumor content on tissue samples, (i) can be subjective, inconsistent and time consuming; (ii) is widely considered to be a bottleneck for many research and diagnostic laboratories; and (iii) if not done precisely, could undermine the reliability of molecular diagnostics.

Percentage tumor nuclei on H&E slides has emerged as one of the key determinants of sample quality and successful molecular testing—as it is currently the most reliable and practical way to determine if there is sufficient tumor DNA in which to detect tumor associated mutations and other genetic aberrations, that may impact on targeted therapy. Depending on the sensitivity of the molecular assay, the minimum percentage of tumor nuclei required in the sample to avoid a false negative result can vary from 1% to 70%. In this current study, we illustrated the subjectivity of tumour annotation and in particular percentage tumor estimations by eye. Reproducibility between pathologists showed gross variation in percentage tumor estimations on lung cancer cases between reads. Some cases varied by as much as 60 points on the % scale and in a majority of cases the percentage of tumour cells was overestimated, with over half of the assessments deviating from ground truth by more than 10 points on the percentage scale. In lung cancer and the molecular evaluation of EGFR mutation for therapeutic selection, inaccurate and overestimated tumor cell content could result in a false nagative test results, potentially leading to anti-EGFR therapy being withheld.

Considerable variation in reporting the % tumor cells in lung tissue samples has been demonstrated in other recent studies in lung [5] and other tissues [6, 28, 29]. Smits et al [5] showed that over a third of lung cancer samples that fell below the crucial 20% limit of detection (LOD) for direct sequencing, were overestimated by pathologists, raising the likelihood of false negative *EGFR* test results in these cases. In colon cancer, a multi-institutional diagnostic trial on percentage tumor estimates for *KRAS* evaluation by Viray *et al*, again showed considerable variation from pathologist to pathologist and similarly suggested that this could have a significant impact on molecular testing for patient stratification and therapy in colorectal cancer [6]. Whilst this is important for patient therapy, this evidence clearly translates across to discovery science and potential flaws in the identification and validation of new mutational targets and biomarkers based on FFPE samples from solid tumors. Clearly a more reliable, quantitative and reproducible method is necessary for estimating tumor cell sufficiency in investigative and diagnostic molecular pathology.

In this study, we developed and validated an automated image analysis method called *TissueMark* for tumor recognition, annotation and analysis which could overcome many of these difficulties. Focusing on applications in non-small cell lung cancer, the method uses high performance image processing, quantitative feature analysis and pattern recognition technology to automatically recognise the tissue patterns associated with lung cancer in H&E tissue scans and differentiate tumor morphology from other non-tumor tissue components. Support Vector Machine technology was used to classify the image using a unique set of image features—a method that has been shown previously to be successful in H&E stained histological images, including cervix [24], breast [30, 31], colon [32] and prostate tissues [33] [36]. Using this approach, the boundary of the tumor can be rapidly computed and presented in a way which can guide laboratory technicians in the macrodissection of samples. Over 97% of cases were shown to be recognised correctly, with statistical analysis of four independent similarity metrics showing strong concordance with conventional hand-drawn boundaries made by experienced pathologists. To further confirm the molecular reliability of automated tumor annotation in this way, a comparison of *EGFR* mutation status between macrodissected samples derived from manual and automatically annotated samples showed the same mutational status with the automated method. While further validation would be necessary, this indicates the potential of using automated imaging tools to support the high throughput analysis of tissue samples in biomarker discovery programs, in patient selection for clinical trials and to support the rapid growth of new molecular tests for primary diagnostics and therapeutic selection.

In addition to automated annotation of tumor boundary to guide macrodissection, the quantitative measurement of percentage tumor cells using the *TissueMark* image analysis method can overcome the shortfalls of visual estimation and provide a more objective measure of tumor cell sufficiency. In order to benchmark this, subjective visual estimations of tumor cell percentage accuracy were not sufficient. Instead we hand-counted a series of tissue images to get precise numbers of tumor and non-tumor cells and an absolute, gold standard measurement of percentage tumor nuclei. Similar types of manual cell counts were carried out by Smit et al [5] and Viray et al [6] but purely for the purposes of comparing against pathological scores. Here, we used this approach to verify accuracy of our image analysis methods and we showed strong concordance between automated image analysis and benchmark nuclear counts. Interestingly, manual bechmark counts took about approximately 4 hours per sample image factoring up to approximately 100 hours per whole slide. Automated image analysis achieved the same results on a whole slide image in under 3 minutes with the same level of precision. This we feel has important potential in measuring sample quality and tumour nucleic acid sufficiency in the molecular evaluation of solid tumors and molecular diagnostics.

Currently, automated boundary generation and % tumor nuclei are separate independent functions. It would however be possible to link them to ensure that defined boundaries only contained tumour cell percentages above defined thresholds, that relate to the limits of detection (LOD) associated with different molecular technologies/biomarkers.

H&E can be a highly variable stain, difficult to control and differs from one laboratory to the next. It is important therefore that any image analysis algorithm that attempts to measure H&E pattern precisely and use this to identify tumor patterns is resilient to stain variability. In this study we explored samples from different laboratories that were stained in different batches where we showed the algorithm to cope with variation in sample preparation and provide reliable results across a range of H&E staining patterns and intensities. The algorithm has been developed to allow the normalization of digital images from different laboratories to ensure consistent interpretation regardless of stain variation. Nevertheless, if the H&E stain falls outside certain expected standards and intensity ranges, tissue recognition ability will be undermined and the algorithm will need to be further adjusted to cope with patterns that emerge from badly stained samples. Nevertheless, the algorithm has been shown to work across a range of laboratory staining practices and can be easily adjusted to fit with the staining preferences of most modern diagnostics and research laboratories.

The tumor identification algorithms were developed initially to operate on the Aperio whole slide imaging platform and the svs image format. Variations in the optics of different scanners, proprietary image formats and image processing methods adopted by hardware manufacturers generally result in images with different colour mixes, contrasts, brightness and backgrounds. Subsequent development of image normalization methodologies which standardises these variables across scanner types has allowed the automated tumor analysis to operate on other whole slide imaging platforms (e.g. Hamamatsu Nanozoomer). Further validation work will be necessary to ensure similar levels of reproducibility, performance and precision on other scanning platforms.

The TissueMark algorithm for lung tumor identification, developed and validated here, has subsequently been integrated into TissueMark workflow software that provides automated markup of tissue samples very rapidly, accelerating pathological review and sample selection within a busy molecular pathology laboratory. In addition to providing the facility to filter and select samples on the basis of objective percentage tumor calculations, the software also prints tumor annotations on paper or acetate sheets in 1:1 slide format for subsequent blank tissue section overlay and macrodissection. This illustrates a real example of translating image analysis technology into a workflow that facilitates the rapid generation of macrodissection boundary markups and quantitative tumor percentage measurements for molecular evaluation in solid tumors.

The methodology presented here is also a generic framework for tissue identification, which could be developed to automatically identify tumor morphology in other histological subtypes in lung cancer and for the automated identification of other tumors types. This approach is facilitated by the widespread use of digital pathology in research and its increasing adoption in diagnostic laboratories. The use of image analysis on whole slide scans for automated tumor markup to guide macrodissection and the measurement of percentage tumor for molecular pathology applications has not been described previously. As a method, it could significantly speed up the turnaround time for macrodissection in large cohort studies or clinical trials facilitating the discovery of molecular biomarkers and integration with other morphomolecular data [34]. It could also improve the objectivity and reliability of tumor markup and percentage tumor estimations for routine molecular diagnostic tests.

In conclusion, this study presents for the first time a powerful image analysis technology which can support tumor markup, sample selection, macrodissection and tumor nucleic acid enrichment in translational molecular research and in molecular diagnostics. Incorporated within dedicated workflow software, this can manage digital slides, track samples, run a range of tumor identification algorithms, store annotations and present these in a lab for physical macrodissection. This has the potential to significantly improve workflow and instil accuracy and consistency in molecular pathology.

## MATERIALS AND METHODS

### Tissue samples

This study was approved by the Office for Research Ethics Committees Northern Ireland (ORECNI). REC reference: 06/NIR01/94. In addition, the molecular evaluation of tissue samples was approved under Northern Ireland Biobank application NIB13-0079.

Samples from 135 NSCLC patients were retrieved from the FFPE archive at Belfast Health and Social Care Trust (BHSCT) by an experienced pathologist, typical of resection cases processed for *EGFR/KRAS* mutation analysis. Sample types included wedge resection, lobectomy and pneumonectomy specimens. Standard laboratory procedures were then followed for FFPE processing for molecular diagnostics. Standard 5 um sections taken from the FFPE blocks were mounted on glass slides and stained with H&E using standard operating procedures adopted in the laboratory. Using a single representative tumor block per case, this provided for the current study a total of 245 H&E slides, of which 110 (44.9%) slides were from non-tumor control FFPE blocks, the remainding 135 (55.1%) showing a mixture of tumor and non-tumor regions.

All slides were scanned using an Aperio ScanScope CS whole slide scanner at 20X magnification using a 20X/0.75 Plan Apo objective. Digital slide images were generated with a resolution of 0.50 μm/pixel and stored in Aperio.svs format. After scanning, these digital slides were compressed using lossy JPEG compression using factory settings on the Aperio platform.

### Manual annotation of slides

A consultant pathologist with a specialist interest in lung cancer reviewed all 245 lung tissue H&E digital slides using PathXL online digital slide viewer (PathXL Ltd, Belfast, UK). In total, the pathologist confirmed 109 slides (44.49%) to be non-neoplastic and contain no tumor tissue. The remaining 136 slides (55.51%) were digitally annotated using the PathXL software to show regions of tumor suitable for macrodissection within the tissue section. The 136 tumor cases comprised a range of histological subtypes including squamous cell carcinoma (SCC), adenocarcinoma, mixed and undifferentiated carcinoma.

### Reproducibility of % tumour cells estimations amongst pathologists

Reproducibility of conventional pathological % tumour estimations was evaluated in two ways. Firstly, the tumor percentages for each of the 136 tumor containing slides were estimated in the overall slide, independently by two different pathologists. Secondly, a series of 20 high resolution images of lung cancers were circulated to 4 pathologists and their independent estimates of % tumour recorded. Eighteen slides were reviewed by all four pathologists, whereas two were reviewed by three pathologists. These were then compared graphically and statistically to determine variability of % lung tumor estimates.

### Manual cell counts for precise % tumor measurements

In order to establish a benchmark dataset for evaluating the precision of tumor cell imaging and automated measurement of percentage tumor cells in in NSCLC, a series of ten cases were randomly selected for manual cell counting. Large 1 mm^2^ regions of the digital slides were randonly located from each of the digital slides and every tumor and non-tumor cells manually counted by hand on the H&E image to derive an accurate percentage tumor figure. Cell counting was facilitated by using the mouse to manually mark the cells on the digital slide. Over 500,000 cells were counted manually for this exercise. All results were reviewed by a second pathologist to ensure that tumor cell identification and manual counts were representative and properly estimated. Whilst time consuming, this provided us with precise benchmark data on the percentage tumor cells in these regions.

### Automated tumor identification using image analysis

#### Training set, feature extraction and classifier

Using expert pathological selection, a training subset of tumor (*n* = 58) and non-tumor (*n* = 46) case images were selected and partitioned into tumor and non-tumor tiles of 31 × 31 pixels at varying resolutions for feature selection and algorithm training. Within this training image set and sub-sample of tumor image regions were selected randomly across the different histological subtypes. Similarly, a sub-sample of non-tumour regions for training included stroma, necrotic tissue, cartilage, normal epithelial tissue, hemorrhagic areas and other areas not consisdered to be tumor tissue. A total number of 50,000 training tiles were created for algorithm training. This was considered to be a minimal training set which could then be applied to the entire series of images, including the set from which the training tiles had been taken from.

A set of 135 image features were extracted from each training tile image. Firstly, the colour image tile was converted into the gray scale and a colour deconvolution method was then used to separate the haematoxylin colour channels from eosin [35]. For these three colour channels, namely haematoxylin, eosin and gray, 45 image features were then calculated, which included 7 histogram features [24, 36, 37], 16 features derived from gray level co-occurrence matrix [24, 38–40], 15 spectral features from the Fourier domain [41] and 7 moment invariant features (invariant to translation, scaling, rotation and mirroring) [42].

**Figure 10:**
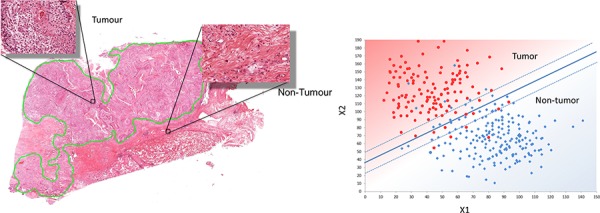
Design of the automated annotation solution **A.** Shows the manual markup and annotatation selection which provides training data for image classification in (B). **B.** Illustrates the linear SVM classifier which can process new images to automatically identify regions of tumour and non-tumour.

A Support Vector Machine (SVM) supervised learning method was constructed and trained to classify tumor and non-tumor tiles using the feature set (Patent application numbers: PCT/GB2014/051426, PCT/GB2014/051427, 9^th^ May 2014). For a 2-class classification, this approach separates feature vectors from 2 categories by obtaining the optimal boundary hyper-plane in a high dimensional space. The popular Gaussian radial basis function was used to fit the maximum margin hyper-plane in a transformed Hilbert space of infinite dimensions [43, 44]. This is illustrated in Figure 10.

After the calculation of features for both tumor and non-tumor tiles, and a grid search 5-fold cross validation, we achieved a strong training accuracy of 89.02%. Similar studies in the literature have reported 87.8% in colon tissue [32], 89.2% in cervical tissue [24] and 96.7% in prostate tissue [45]. The training accuracy is dependent on the selection of multiple factors including image tile dimensions, feature sets and supervised learning methods. The trained support vector model was obtained which could optimally detect tumor from other digital slides.

#### Automated analysis of tumor nuclei counts and tumor percentage

Using image analytics and tissue recognition, a number of methods were devised to calculate the percentage of tumor nuclei within the tissue sample. These ranged from calculating the percentage of image tiles that were classed as tumour through to the estimation of actual tumor cell number within those tissue tiles. Since image tiles can contain different densities of tumor, it was considered that tissue area estimation would not offer the precision necessary for assessing molecular nucleic acid quality. Whole slide scans on the Aperio platform provided resolutions up to 0.24 um per pixel allowing high resolution imaging and analysis of nuclear content. Following adaptive thresholding of the image, nuclear segmentation allowed an evaluation of global nuclear content followed by estimation of nuclei numbers based on nuclear size modelling (Patent application numbers: WO2014/184522 and WO2014/181123). This provided a rapid and efficient method of estimating nuclear numbers in both the tumor and non-tumor regions without the need for detailed individual nuclei segmentation. Cell counts and percentage tumor estimations were subsequently compared with manual hand-counted benchmark data to determine accuracy.

#### Testing

**Figure 11:**
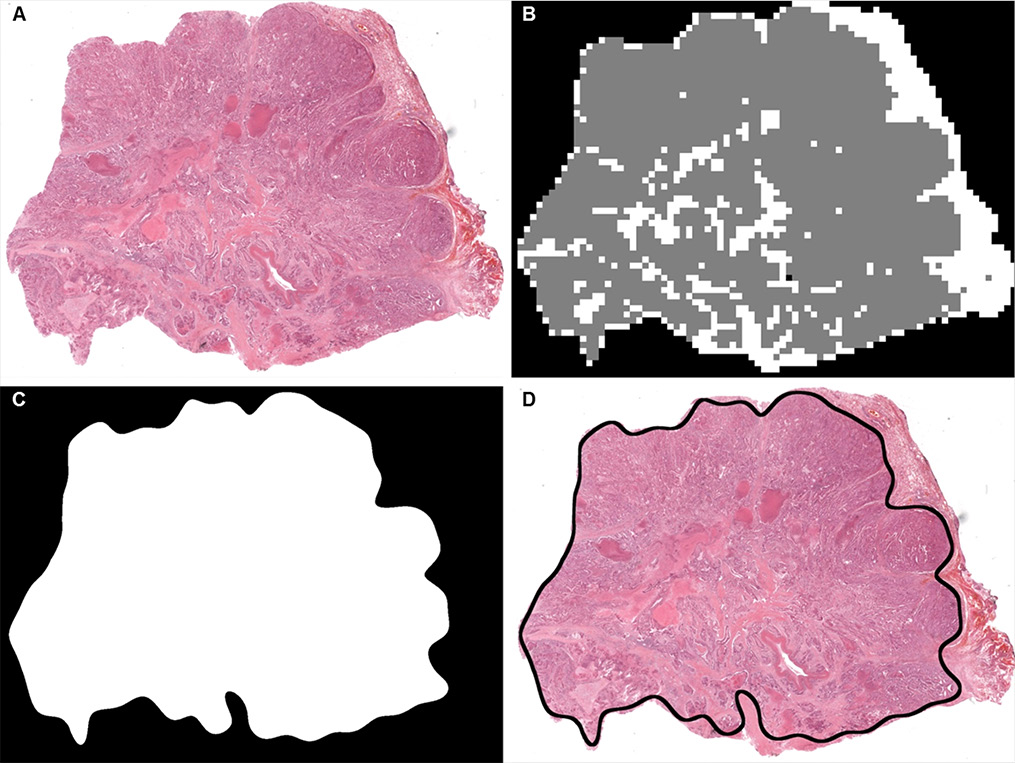
An example showing how a tissue slide is annotated using the automatic tissue identification **A.** An original H&E stained lung tissue slide captured. **B.** A pseudo coloured *TumorMap* image after processing, where gray represents tumor rich tissue, white represents non-tumor tissue and black shows the white background (void). **C.** A refined *TumorMap* image where the boundary has been smoothed to support tissue annotation. **D.** An overlay of the *TumorMap* boundary on top of the original lung slide.

The tumour identification and analysis algorithm developed above was subsequently applied to whole slide digital images in the series. For a new whole slide H&E lung cancer scan, the background was firstly recognised and removed using a simple Otsu's thresholding method [41], The remaining tissue components were then partitioned into 31 × 31 pixel tiles at a range of resolutions. As with the training process, a set of 135 image features were then calculated for each tile. Using the trained support vector model, each tile was classified as tumor or non-tumor. The tile label was then used to construct a spatial map of the image, in which the background in the original tissue slide (Figure 11A) is marked in black, tumor regions are marked in gray and non-tumor regions are marked in white in the image map (Figure 11B). The image is further processed to remove isolated regions using image processing techniques including image opening and closing, and hole filling. The irregular boundaries were then smoothed using a pair of forward and inverse Fourier descriptors. Smoothing was important to ensure that the resulting image could be used as a practical mask for macrodissection. Finally the refined tumor boundary annotation was produced, as in the example shown in Figure 11C & 11D.

A posterior probability in the range of [0–1] was also computed for each tile to indicate the likelihood for tumor or non-tumor class membership. This could be presented as a *TumorMap*, allowing the visualisation of tumor regions color coded as the probability of tumor.

### Statistical evaluation of algorithm performance

It was important to compare manually drawn tumor boundaries with boundaries generated using automated image analysis. Visual assessment by an experienced pathologist allowed us to determine the quality and accuracy of automated tumor annotation as well as a visual comparison between manual and automated annotation and an evaluation on whether deviations would have impact on molecular testing. These were largely subjective evaluations. In addition, however, we devised four methods to measure boundary differences: (i) inclusion rate vs. exclusion rate, (ii) receiver operating characteristic (*ROC*) curve, (iii) concordance index and (iv) false discovery rate (*FDR*). These are fully described in Appendix 1. In addition, tumor nuclear count data and percentage tumor cells derives using the algorithm was compared against the gold standard benchmnark of hand counts on a series of high resolution images.

### Molecular evaluation

In order to evaluate the consistency of molecular test results from manually and automatically annotated tissues samples, a series of 6 lung cancer clinical cases were randomly selected from our molecular diagnostic service (Northern Ireland Molecular Pathology Laboratory, holding CPA accreditation), previously genotyped for *EGFR* mutation status. These had been previously macrodissected using a manual boundary to enrich the sample, followed by DNA extraction using the Roche COBAS DNA Preparation Kit and *EGFR* mutation status determined by Roche COBAS EGFR IVD kit using COBAS 4800 LightCycler software. New sequential sections were taken from the tissue block. Following H&E staining of one section, the slide was digitally scanned as previously described and subjected to the tumor identification algorithm. The digitally annotated images were used as the guide for subsequent macrodissection and the enriched sample subjected to DNA extraction and *EGFR* mutation evaluation.

## APPENDIX 1

Firstly, computer generated tumor boundaries were overlaid with hand drawn annotations, and level of overlap was measured using a vraiety of approaches. True positive (*TP*) areas were pixels where the manual and automated methods agreed on identification of tumor. True negative (*TN*) regions were pixels where the manual and automated methods agreed on identification of non-tumor. False positive (*FP*) was defined as the number of pixels annotated as non-tumor by the pathologist but identified as tumor by the automated method. False negative (*FN*) was defined as the number of pixels annotated as tumor by the pathologist but identified as non-tumor by the automated method. White background (void) is excluded from all calculations. To further validate tumor boundary concurrence, we defined *inclusion rate* to be the sensitivity (*SEN)* of overlapping tissue area, and *exclusion rate* to be the specificity (*SPE)* of overlapping tissue area. These two values are in the numerical range of [0–100%]. The higher these two values are, the more similar the automated annotation is to a pathologist's manual annotation. Finally, we also generated a ROC curve and its area under the curve (*AUC*) value as a measure of accuracy, demonstrating the discriminating power.

The above two measurements, *inclusion rate* vs. *exclusion rate* and ROC curve have similar weightings when assessing boundary concordance. In macrodissection however, the impact of false positive is greater as it is more important to exclude non-tumour material from the boundary. Therefore, we introduced a new numerical measurement namely concordance index (*CI*). *CI* is in fact the *F_β_* score with *β* < 1 to highlight false positives embedded in the precision measurement (48).

In this study, we use *CI* = *F*_0.5_ to measure the effectiveness of the proposed automatic tumor identification therefore placing half as much importance on sensitivity as precision (positive predictive value).

To further explore false positive cases, we also used false discovery rate (*FRD*) to highlight the worst false positive cases:

(1) $$\text{FDR}=\frac{N(\text{FP})}{N(\text{TP})+N(\text{FP})}$$

where N(▪) is the number of pixels inside a region. *FDR* is in the range of [0–1] and clearly the smaller the *FDR* value, the better the performance.
